# Time-Course Transcriptomic Analysis of Early Host Responses to Oral SfMNPV Challenge in *Spodoptera frugiperda* Larval Midgut

**DOI:** 10.3390/insects17040401

**Published:** 2026-04-08

**Authors:** Lin Guo, Wenyi Jin, Yan Tong, Huixian Shi, Qin Kang, Jihong Zhang, Qian Meng, Xuan Li, Hongtuo Wang, Qilian Qin, Huan Zhang

**Affiliations:** 1 State Key Laboratory of Animal Biodiversity Conservation and Integrated Pest Management, Institute of Zoology, Chinese Academy of Sciences, Beijing 100101, China; guolin@ioz.ac.cn (L.G.); jinwenyi23@ioz.ac.cn (W.J.); tongy@ioz.ac.cn (Y.T.); 20238017001@stumail.hbu.edu.cn (H.S.); kangqin22@ioz.ac.cn (Q.K.); zhangjh@ioz.ac.cn (J.Z.); mengqian@ioz.ac.cn (Q.M.); lix@ioz.ac.cn (X.L.); wanght@ioz.ac.cn (H.W.); 2 University of Chinese Academy of Sciences, Beijing 101408, China

**Keywords:** *Spodoptera frugiperda*, SfMNPV, transcriptome, midgut

## Abstract

The fall armyworm (*Spodoptera frugiperda*) is a major global migratory pest. Its increasing insecticide resistance poses a severe threat to food security. Developing biopesticides such as SfMNPV is critical for sustainable control. Nevertheless, the early molecular mechanisms underlying the *S. frugiperda* midgut response to oral SfMNPV challenge remain poorly understood. This study utilized high-throughput transcriptome sequencing to systematically characterize the dynamic transcriptional profiles of the larval midgut at 1, 12, and 24 h after oral SfMNPV inoculation. Results showed that the midgut transcriptional response to SfMNPV is time and stage-specific. During this period, the physical midgut barrier underwent remodeling, with core components of the peritrophic matrix downregulated at 1 h, followed by the basal lamina at 12 h, alongside the activation of cytoskeleton genes during 12–24 h. Concurrently, sustained endoplasmic reticulum stress, autophagy, and ubiquitin system responses occurred from 12 to 24 h. At the metabolic level, the defense system exhibited a functional succession, shifting from ABC transporters and UDP-glycosyltransferases at 1 h to glutathione S-transferases and superoxide dismutase at 12–24 h. Additionally, the midgut tissue exhibited a cascade transition from pro-apoptotic signaling at 1 h to compensatory regenerative repair mediated by the Wnt, mTOR, and Hippo pathways at 12–24 h. This study elucidates the molecular process of barrier damage, homeostatic imbalance, and tissue remodeling during early oral SfMNPV challenge. These findings provide a global perspective on baculovirus-host interactions and establish a theoretical foundation for designing novel biopesticides targeting the midgut interaction.

## Simple Summary

To combat the escalating insecticide resistance of the fall armyworm (*Spodoptera frugiperda*), Spodoptera frugiperda multiple nucleopolyhedrovirus (SfMNPV) serves as a sustainable biological alternative. However, the early responses of the larval midgut to oral SfMNPV challenge remain unclear. We performed time-course transcriptome sequencing to systematically characterize the host’s molecular dynamics during viral challenge. Our findings reveal a phased transcriptional progression in the midgut at 1, 12, and 24 h after oral SfMNPV inoculation. During these stages, the midgut barrier was damaged and accompanied by cytoskeleton reorganization. Simultaneously, sustained protein-processing stress and cellular degradation pathways were induced. Host biochemistry shifted from detoxification metabolism to antioxidant defense, while the midgut tissue underwent a cascade transition from cell apoptosis to tissue regeneration. This research elucidates the complex warfare between the virus and its host, providing a strategic roadmap for engineering superior biopesticides that exploit specific vulnerabilities in the insect gut.

## 1. Introduction

The fall armyworm, *Spodoptera frugiperda* (Lepidoptera: Noctuidae), is a native agricultural pest insect in the Americas [1]. Since its initial detection in Africa in 2016, it has emerged as one of the most destructive invasive pests in global agricultural production [2]. With a broad host range, formidable migratory capacity, and high reproductive potential, *S. frugiperda* can trigger rapid outbreaks and pose a sustained threat to staple crops, including maize and other cereals [3]. Currently, field management still relies heavily on chemical insecticides and Bt crops. However, the high adaptability of *S. frugiperda*, coupled with the selective pressure imposed by frequent insecticide applications, has led to the emergence and accumulation of insecticide resistance across multiple regions [4,5,6,7]. This escalating resistance complicates management efforts and highlights the urgent need for more eco-friendly management strategies.

In this context, baculovirus-based biopesticides have gained significant attention due to their high host specificity and favorable environmental safety profile [8]. Spodoptera frugiperda multiple nucleopolyhedrovirus (SfMNPV), a specialist baculovirus of *S. frugiperda*, has been registered as a commercial formulation in various countries and regions [9]. As a critical component of eco-friendly pest control measures, SfMNPV represents a key biological tool for mitigating the impact of *S. frugiperda*. Baculoviruses are a group of arthropod-specific large double-stranded DNA viruses [10]. Their infection cycle initiates when the host ingests occlusion bodies (OBs), which dissolve in the alkaline midgut and release occlusion-derived virions (ODVs). These ODVs traverse the peritrophic matrix (PM) and fuse with the microvilli of midgut epithelial cells, delivering the viral genome to the nucleus to initiate primary infection. Subsequently, progeny budded virions (BVs) cross the basal lamina and enter the hemolymph, thereby establishing widespread systemic infection [11]. Consequently, successful traversal of the intestinal barrier is a major determinant of viral replication efficiency and overall pathogenicity within the host.

The insect gut deploys a coordinated defense system against baculovirus invasion. This defensive framework primarily relies on the structural integrity of physical barriers, including the PM, the epithelial layer, and the basal lamina [12,13,14]. Biochemical defenses include the activation of detoxification and antioxidant enzymes, along with the secretion of digestive enzymes [15,16,17]. Furthermore, the gut activates a broad range of immune and stress-response pathways, including Toll, IMD, JAK/STAT, JNK, RNA interference, and apoptosis [18,19,20,21]. Correspondingly, baculoviruses have evolved various antagonistic strategies to counteract host defenses. Typical mechanisms include degrading the PM to breach this physical barrier [22,23]. In addition, baculovirus midgut infection is associated with changes in host restriction programs, including pathways related to apoptosis and melanization, and with broad transcriptional and metabolic reprogramming [18,24,25]. Together, these interactions define the early phase of midgut infection as a critical window period. At this stage, despite the absence of massive viral amplification, the host initiates a rapid and extensive reprogramming of its intestinal barriers, signaling pathways, and immune networks [18,26]. Therefore, elucidating these dynamics and the host–virus antagonism is essential for enhancing the efficacy and stability of baculovirus-based biopesticides.

Although transcriptomic changes during primary baculovirus infection have been explored in *S. frugiperda* using a subtractive suppression cDNA library approach, time-course RNA-seq characterization of larval midgut responses to oral SfMNPV challenge during this critical early window remains limited [27]. Given these limitations, we performed midgut transcriptomic profiling of *S. frugiperda* at 1, 12 and 24 h after oral SfMNPV inoculation. This study characterizes dynamic host response patterns and identifies candidate genes with potential value for pest control. Beyond advancing the understanding of baculovirus-insect interactions, our findings provide a molecular basis for developing integrated pest management (IPM) strategies against insecticide-resistant *S. frugiperda* larvae.

## 2. Materials and Methods

### 2.1. Insects and Viruses

The *S. frugiperda* population used in this study was originally collected from maize fields in Guangdong Province, China. The insects were reared on an artificial diet (prepared according to Shu et al. [28]) for over 30 consecutive generations under laboratory conditions to establish a stable colony. Rearing conditions were 27 ± 1 °C, 70 ± 5% relative humidity, and a 12:12 h light: dark photoperiod. A purified SfMNPV stock (CCTCC No. V202062) was provided by Henan Jiyuan Baiyun Industry Co., Ltd. (Jiyuan, China). After quantification, the virus was diluted to a final concentration of 1.0 × 10^8^ occlusion bodies (OBs)/mL and stored at −20 °C.

### 2.2. Oral Inoculation and Bioassay

To ensure experimental consistency, healthy and developmentally synchronized larvae were used. Late third-instar larvae approaching ecdysis were starved for 12 h, and newly molted fourth-instar larvae were selected for the bioassay. For oral inoculation, the artificial diet was cut into standardized small cubes of approximately equal size, and 1 μL of virus suspension was pipetted onto each cube. Each larva was individually provided with one treated diet cube and allowed to feed for 10 min. Only larvae that completely consumed the entire cube were retained for subsequent experiments. Larvae in the control group were fed diet cubes treated with an equal volume of ddH_2_O. Each group comprised 60 larvae. After dosing, all individuals were transferred to a virus-free artificial diet for continued rearing. Larval survival was monitored daily, and dead individuals were recorded and removed promptly. Observations continued until all larvae either pupated or died. During inoculation and the subsequent rearing period, all larvae were maintained under the same environmental conditions as the stock colony (27 ± 1 °C, 70 ± 5% relative humidity, and a 12:12 h light:dark photoperiod).

### 2.3. Sample Collection and RNA Extraction

For transcriptome analysis, midguts were collected at 1, 12, and 24 h after oral SfMNPV challenge described above, and ddH_2_ O-fed larvae were collected in parallel at each time point as controls to mitigate developmental effects on gene expression. The midguts were cleared of contents, snap-frozen in liquid nitrogen, and stored at −80 °C until RNA extraction. Three biological replicates were prepared per time point, and each replicate consisted of pooled midguts from 10 larvae. Total RNA was extracted from the samples using TRIzol reagent (Thermo Fisher Scientific, Waltham, MA, USA) according to the manufacturer’s instructions. Quantity and purity of RNA were measured using a NanoDrop 2000 spectrophotometer (Thermo Fisher Scientific, Waltham, MA, USA), while integrity was assessed by agarose gel electrophoresis and an Agilent 5300 system (Agilent Technologies, Santa Clara, CA, USA). High-quality RNA was defined as having a total amount of approximately 1 μg, a concentration ≥ 30 ng/μL, an RQN > 6.5, and an OD_260_/OD_280_ ratio between 1.8 and 2.2. All samples met these criteria for library construction and sequencing.

### 2.4. Library Construction, Sequencing, and RNA-Seq Data Processing

The transcriptome libraries were constructed from 1 μg total RNA using the Illumina Stranded mRNA Prep Ligation Kit (Illumina, San Diego, CA, USA). Briefly, poly(A) mRNA was enriched and fragmented (~300 bp) for first- and second-strand cDNA synthesis. The resulting fragments underwent end-repair, 3′ A-tailing, and adapter ligation. Final libraries were amplified by PCR and sequenced on the Illumina NovaSeq X Plus platform (Illumina, San Diego, CA, USA) at Shanghai Majorbio Bio-pharm Technology Co., Ltd. (Shanghai, China). Raw FASTQ reads were processed using fastp (v0.23.4) to obtain clean reads by removing adapters and low-quality sequences. The quality of the clean reads was assessed based on GC content, Q20, Q30, and base error rates. The clean reads were then aligned to the *S. frugiperda* reference genome (NCBI RefSeq assembly: GCF_023101765.2) and SfMNPV reference genome (NCBI RefSeq assembly: GCF_000868825.1) using HISat2 (v2.2.1). Transcript assembly was performed using StringTie (v2.2.1), and functional annotation was achieved by aligning the assembled sequences against the NR (v2023.07), Swiss-Prot (v2023.11), Pfam (v36.0), eggnog (v2020.06), GO (v2023.07) and KEGG (v2023.09) databases.

### 2.5. Differential Expression Analysis

Gene expression levels were quantified using RSEM (v1.3.3) based on the alignment results, generating TPM (transcripts per million) and raw read counts for downstream analyses. Principal component analysis (PCA) was performed to assess biological reproducibility among samples. Differential expression analysis was conducted using DESeq2 (v1.56.1), with genes having Padj < 0.05 and |log2 FC| ≥ 0.585 defined as differentially expressed genes (DEGs). UpSet plots were used to visualize the intersection and unique distribution of DEGs across treatment groups. DEGs were further grouped according to expression clusters identified using custom R scripts. Functional enrichment of DEGs in each cluster was performed using GO and KEGG annotations. GO enrichment was conducted with Goatools (v1.4.4), and KEGG pathway enrichment was performed using the R clusterProfiler package (v4.10.0). *p*-values from GO and KEGG enrichment analyses were adjusted for multiple testing using the Benjamini–Hochberg method, and terms/pathways with Padj < 0.05 were considered significantly enriched.

### 2.6. RT–qPCR Validation

To validate the reliability of the RNA-seq data, the relative expression levels of candidate genes were determined via quantitative real-time PCR (RT-qPCR). The ribosomal protein L13 gene (RPL13) was utilized as the internal reference for normalization. Gene-specific primers were designed using Primer Premier 6.0 (Table S1). Total RNA (1 μg) was reverse-transcribed into cDNA using the Evo M-MLV RT Mix Kit with gDNA Clean for qPCR Ver. 2 (Accurate Biology, Changsha, China) according to the manufacturer’s instructions. The qRT-PCR assays were conducted using the SYBR Green Premix Pro Taq HS qPCR Kit (Accurate Biology) on a QuantStudio 12K Flex Real-Time PCR System (Applied Biosystems, Foster City, CA, USA). The reaction components and thermal cycling conditions strictly followed the manufacturer’s protocols. Each treatment consisted of three biological replicates, each performed with three technical replicates.

### 2.7. Data Analysis

Statistical analyses were performed using GraphPad Prism 7.00. For bioassays, survival rates were compared using the log-rank (Mantel–Cox) test to generate survival curves. For RT-qPCR, relative gene expression was calculated using the 2^−ΔΔCt^ method, normalized to the RPL13 gene. Significant differences between groups were evaluated using a two-tailed Student’s *t*-test. Statistical significance was defined at *p* < 0.05.

## 3. Results

### 3.1. Quality Assessment and Mapping of Transcriptomic Data

**Figure 1:**
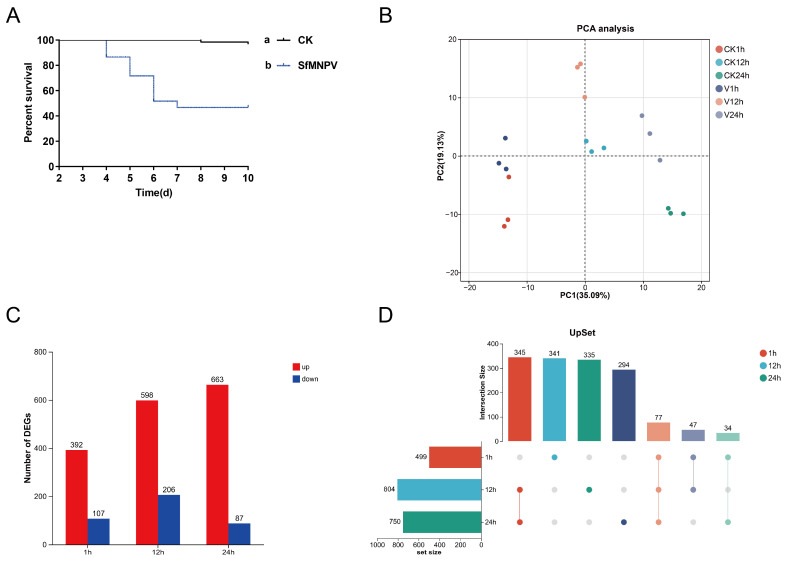
Global transcriptional response of *Spodoptera frugiperda* midgut to oral SfMNPV challenge. (**A**) Bioassay results (survival curves) of *S. frugiperda* larvae following oral SfMNPV challenge (n = 60). Different lowercase letters indicate significant differences between groups based on the log-rank test (*p* < 0.05). (**B**) Principal component analysis (PCA) plot of all transcriptome samples based on gene expression levels. (**C**) Total number of DEGs at 1, 12, and 24 h. (**D**) UpSet plot showing the intersection and specific DEGs at 1, 12, and 24 h. CK: control group; V: SfMNPV-challenged group.

In this study, fourth-instar *S. frugiperda* larvae were orally challenged with SfMNPV, inducing approximately 50% mortality (Figure 1A). This experimental design aimed to capture key host factors involved in early antiviral defense and tolerance regulation without inducing rapid, mass mortality. Transcriptome sequencing was performed on 18 midgut samples, yielding 40,106,832 to 54,164,494 clean reads (approximately 6.03 to 8.14 Gb) per sample after quality control. Data assessment showed base error rates of 0.0118–0.0121%, Q20 levels of 99.12–99.27%, Q30 levels of 95.54–96.27%, and GC content of 47.64–50.2%, confirming high data reliability (Table S2).

The mapping rate of each sample to the *S. frugiperda* reference genome ranged from 84.77% to 87.0%. Concurrently, mapping to the SfMNPV genome (143 genes) revealed that viral read counts in the SfMNPV-challenged groups were generally low. Viral reads per sample ranged from 0 to 637, representing a minimal proportion of total reads (maximum < 0.002%), which was consistent with an early stage of viral activity and a low viral load. SfMNPV exhibited progressive transcriptional activation in the midgut, with negligible activity at 1 h but significant increased at 12 and 24 h, accompanied by notable inter-individual variation and strong biological heterogeneity (Table S3).

### 3.2. Global Transcriptional Response

To evaluate the global transcriptional changes in the *S. frugiperda* midgut following oral SfMNPV challenge, principal component analysis (PCA) was performed on samples (Figure 1B). The results showed clear spatial distribution patterns, with biological replicates clustering tightly, indicating high data consistency. PC1 accounted for 35.09% of the total variance, with a distinct separation of samples reflecting a strong time-dependent shift in midgut transcription. PC2 accounted for 19.13% of the total variance, further capturing the impact of oral SfMNPV challenge on the transcriptional profile. At identical time points, the SfMNPV-challenged and control groups showed clear divergence along the PC2 axis, which became more pronounced at 12 and 24 h. This indicates that viral challenge further drives the divergence of the midgut transcriptome, with the effect intensifying over time.

A total of 17,923 genes were identified, including 16,908 reference genes and 1015 novel genes. Differential expression analysis revealed 499 DEGs at 1 h (392 up, 107 down), 804 DEGs at 12 h (598 up, 206 down), and 750 DEGs at 24 h (663 up, 87 down). Overall, the virus-induced transcriptional response peaked at 12 h and remained substantial at 24 h, primarily characterized by gene upregulation (Figure 1C). UpSet analysis revealed that only 77 DEGs were shared across all three time points, indicating a highly stage-specific transcriptional response in the midgut. The majority of transcriptional changes were not sustained across all stages, with a substantial number of stage-specific DEGs identified at each time point. Specifically, 341, 335, and 294 unique DEGs were identified at 1, 12, and 24 h, respectively. The highest proportion of unique DEGs occurred at 1 h, suggesting a robust transient response during the early stage. The number of shared DEGs between 12 and 24 h reached 345, reflecting a more continuous and stable host response over time (Figure 1D).

### 3.3. Temporal Expression Patterns and Functional Enrichment of DEGs

**Figure 2:**
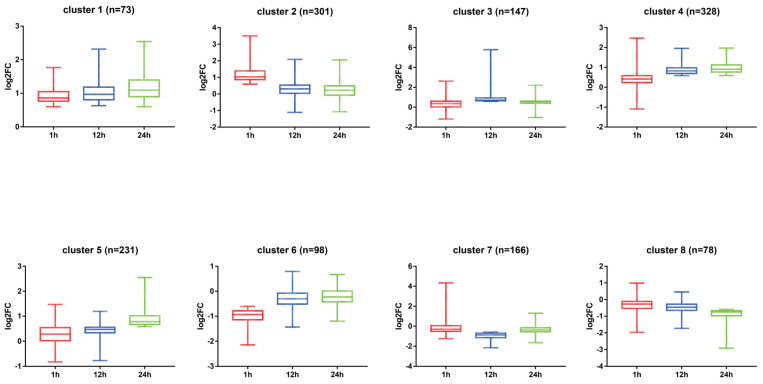
Dynamic clustering analysis of DEGs in the *S. frugiperda* midgut following oral SfMNPV challenge. All DEGs were classified into eight clusters (Clusters 1–8) based on their expression trends at 1, 12, and 24 h. cluster 1: 1–24 h persistent upregulation; cluster 2: 1 h specific upregulation; cluster 3: 12 h specific upregulation; cluster 4: 12–24 h persistent upregulation; cluster 5: 24 h specific upregulation; cluster 6: 1 h specific downregulation; cluster 7: 12 h specific downregulation; cluster 8: 12–24 h continuous downregulation.

**Figure 3:**
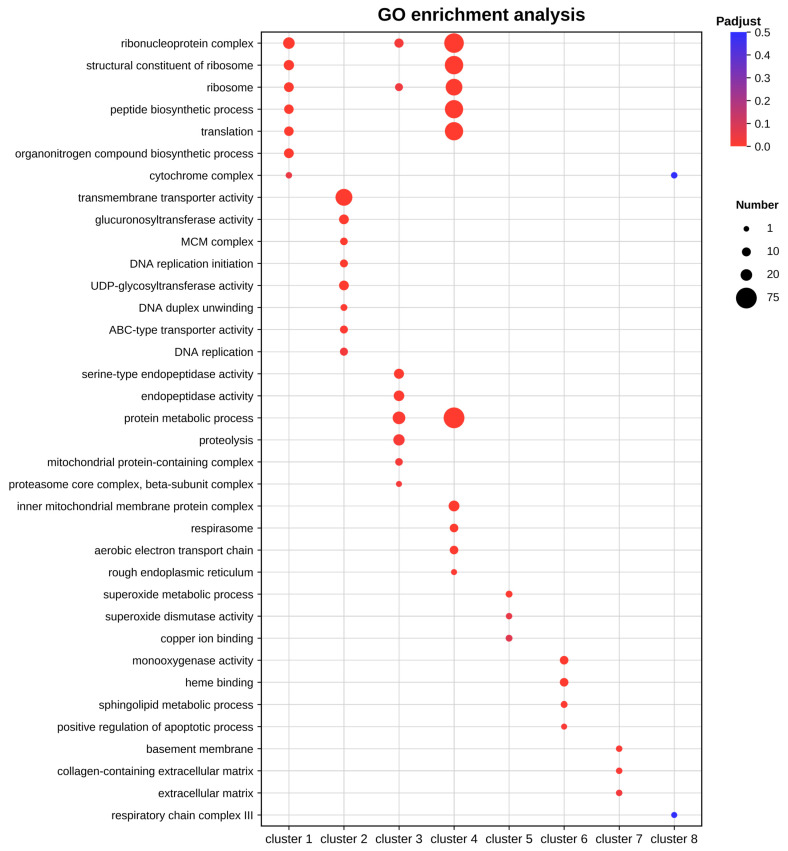
GO functional enrichment analysis of DEGs in different clusters.

**Figure 4:**
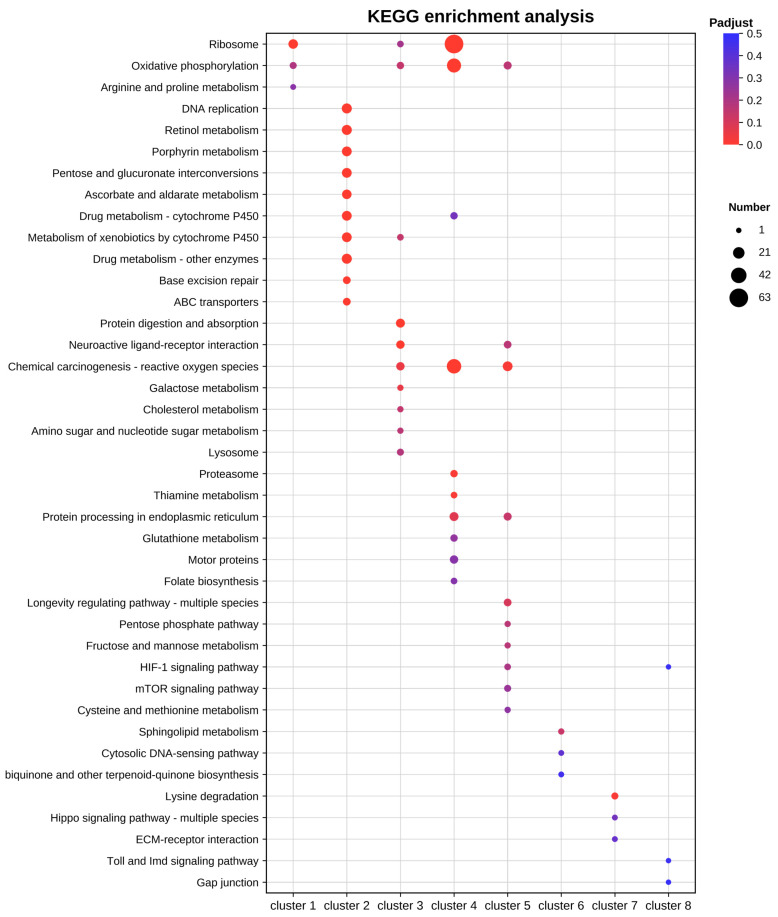
KEGG pathway enrichment analysis of DEGs in different clusters.

To analyze the temporal dynamics of the midgut transcriptional response, DEGs were categorized into eight representative patterns based on their expression trends at 1, 12, and 24 h (Figure 2). Subsequently, GO functional (Figure 3) and KEGG pathway (Figure 4) enrichment analyses were performed for each cluster. Results showed that upregulated patterns dominated the host response and exhibited clear stage-dependent characteristics.

In the persistent upregulation patterns, genes in cluster 1 remained upregulated throughout the entire period. These genes were significantly enriched in ribosome and translation pathways. This suggests that a few core genes may maintain basic physiological homeostasis and stress resistance by sustaining high levels of protein synthesis. Cluster 4 showed continuous upregulation from 12 to 24 h. Its functions involved peptide biosynthetic processes, oxidative phosphorylation, and chemical carcinogenesis-reactive oxygen species pathway. This implies that the host synchronized energy metabolism and protein synthesis to compensate for energy loss during these stages.

Regarding stage-specific upregulation, cluster 2 showed features of genome stability maintenance and detoxification at 1 h. This cluster was significantly enriched in DNA replication and MCM complex, suggesting the host was actively responding to viral replication pressure. Meanwhile, enrichment in cytochrome P450 pathways, transmembrane transporter activity, and UDP-glycosyltransferase activity indicated that the host rapidly activated detoxification and transport systems. At 12 h, cluster 3 exhibited significant metabolic reprogramming. The DEGs were primarily enriched in protein digestion and absorption, as well as endopeptidase activity. This suggests the host enhanced digestive and absorptive functions to meet the energy demands of antiviral defense. At 24 h, cluster 5 was significantly enriched in superoxide metabolic process and chemical carcinogenesis-reactive oxygen species pathway. This indicates that an enhanced oxidative stress response was the primary physiological feature of the midgut at the late stage.

In contrast, the enrichment features of the downregulated patterns were generally weaker. Cluster 6 showed specific downregulation at 1 h, mainly involving monooxygenase activity and heme binding. Notably, cluster 7 showed specific downregulation at 12 h, with significant enrichment in the basal lamina and extracellular matrix. This suggests that the integrity of the midgut structure and biological barrier may be affected by transcriptional downregulation. For cluster 8, which was consistently downregulated from 12 to 24 h, no significantly enriched pathways were detected.

### 3.4. Temporal Remodeling of Midgut Barriers

**Figure 5:**
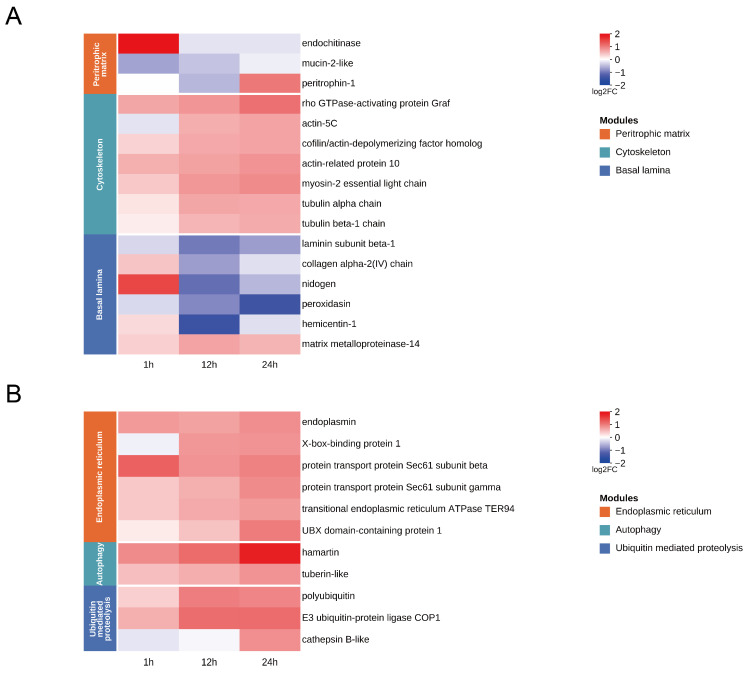
Temporal expression patterns of DEGs in key functional modules of *S. frugiperda* midgut after oral SfMNPV challenge. (**A**) Heatmap of DEGs related to midgut barriers. (**B**) Heatmap of DEGs related to endoplasmic reticulum stress, autophagy, and protein homeostasis.

Midgut structural barrier genes in *S. frugiperda* exhibited temporal changes following SfMNPV challenge (Figure 5A). At 1 h, genes related to the PM responded rapidly. Specifically, *mucin-2-like* was downregulated, while *endochitinase* was upregulated. By 24 h, PM structural protein *peritrophin-1* was upregulated. This indicated that the PM structure underwent remodeling during the viral challenge. Concurrently, genes associated with the cytoskeleton and morphological regulation showed a continuous upward trend. Cytoskeletal and motor proteins, including *actin*, *myosin*, and *tubulin*, as well as *Graf*, were upregulated between 12 and 24 h. The activation of *Graf* as early as 1 h suggested that SfMNPV induced cytoskeletal rearrangement and enhanced cell dynamics. At the basal lamina level, highly consistent transcriptional changes occurred at 12 h. Multiple key component and cross-linking genes, including *laminin subunit beta-1*, *collagen alpha-2(IV) chain*, *nidogen*, *peroxidasin*, *hemicentin-1*, were downregulated. Meanwhile, *matrix metalloproteinase -14* was upregulated. These shifts suggested that basal lamina remodeling during this period potentially affected epithelial barrier stability and cell adhesion.

### 3.5. Endoplasmic Reticulum Stress, Autophagy, and Protein Homeostasis

Host pathways related to endoplasmic reticulum stress, autophagy, and ubiquitin-mediated proteolysis were extensively activated after SfMNPV challenge, showing distinct temporal expression patterns (Figure 5B). At 1 h, the endoplasmic reticulum-specific molecular chaperone *endoplasmin* was activated and remained highly expressed until 24 h, suggesting that SfMNPV induces persistent protein folding stress. From 12 to 24 h, key endoplasmic reticulum stress regulators, including *X-box-binding protein 1*, *protein transport protein Sec61 subunit beta*, *protein transport protein Sec61 subunit gamma*, *transitional endoplasmic reticulum ATPase TER94*, and *UBX domain-containing protein 1*, were continuously upregulated. Meanwhile, autophagy initiation factors such as *hamartin* and *tuberin-like*, along with key ubiquitin pathway genes including *polyubiquitin* and *E3 ubiquitin-protein ligase COP1*, exhibited coordinated upregulation. This phenomenon suggests that host cells undergo systemic adjustments in protein processing and degradation. At 24 h, the lysosomal protease *cathepsin B-like* was further activated, potentially reflecting intensified intracellular degradation and membrane system remodeling.

### 3.6. Detoxification Metabolism and Redox Homeostasis

**Figure 6:**
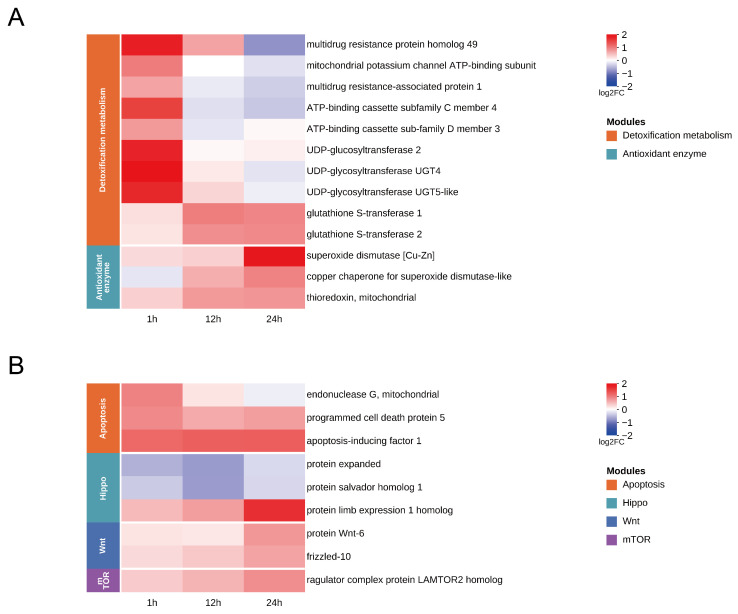
Temporal expression patterns of DEGs in key functional modules of *S. frugiperda* midgut after oral SfMNPV challenge. (**A**) Heatmap of DEGs related to detoxification metabolism and redox homeostasis. (**B**) Heatmap of DEGs related to apoptosis and regeneration of midgut epithelial cells.

During the early stage of SfMNPV challenge in the *S. frugiperda* midgut, host cells rapidly initiated defense responses centered on metabolic detoxification and membrane transport (Figure 6A). Transcriptome data revealed significant upregulation of several ATP-binding cassette (ABC) transporter genes at 1 h. Simultaneously, the *UDP-glycosyltransferase genes UGT2*, *UGT4*, and *UGT5* were also significantly upregulated. As the response progressed to 12 h, the host defense response evolved further, with *glutathione S-transferase* being significantly upregulated, a trend that persisted until 24 h. At 24 h, host cells activated a key antioxidant enzyme system. Key enzymes, including *superoxide dismutase [Cu-Zn]*, its molecular chaperone *copper chaperone for superoxide dismutase*, and *thioredoxin*, were significantly upregulated. This suggests that oxidative stress induced by viral replication was further exacerbated in midgut cells, prompting the host to initiate corresponding defense mechanisms.

### 3.7. Apoptosis and Regeneration of Midgut Epithelial Cells

Oral SfMNPV challenge in the *S. frugiperda* midgut triggered gene expression fluctuations, transitioning from stress-induced apoptosis to compensatory regeneration (Figure 6B). At 1 h, the mitochondrial-mediated cell death program was activated, evidenced by the significant upregulation of mitochondrial *endonuclease G* and *programmed cell death protein 5*. Furthermore, *apoptosis-inducing factor 1* remained consistently upregulated at 1, 12, and 24 h, indicating that apoptosis is the primary physiological response to viral challenge. At 12 h, the core inhibitory components of the Hippo pathway, *protein expanded* and *protein salvador homolog 1*, were significantly downregulated, suggesting the removal of restrictions on stem cell proliferation. By 24 h, the midgut exhibited a trend toward comprehensive repair and renewal, characterized by the synchronous upregulation of genes across multiple core signaling pathways. These genes included *protein Wnt-6* and *frizzled-10* of the Wnt pathway, the *ragulator complex protein LAMTOR2 homolog* in mTOR signaling, and the *protein limb* expression 1 homolog in the Hippo pathway. The synergistic activation of these genes suggests that the host has fully initiated compensatory repair and tissue regeneration of the damaged epithelium.

## 4. Discussion

Our results suggest that the *S. frugiperda* midgut exhibits a dynamic and stage-specific transcriptional response following oral SfMNPV challenge. Rather than representing a static pattern, this response appears to follow a clear temporal progression, indicating that the host may sense viral invasion early and coordinate molecular responses to cellular stress. In this study, we used a sublethal oral challenge causing approximately 50% mortality in order to preserve the physiological integrity of the midgut, thereby allowing more accurate capture of early defense and adaptive responses. At the same time, this sublethal exposure was also accompanied by noticeable transcriptional variation among biological replicates. Such variation is likely a common feature of in vivo oral infection models, in which infection progression may not be fully synchronized among individuals [29]. In addition, single-nucleus sequencing has shown that cellular heterogeneity in the midgut can also influence transcriptional profiles, suggesting that part of the observed variation may reflect the inherent complexity of baculovirus-host interactions [30].

### 4.1. Dynamic Remodeling of Midgut Structural Barriers During Baculovirus Infection

During baculovirus oral infection, the viruses must traverse multiple midgut barriers to enter the hemocoel and subsequently spread to other tissues. The PM composed of chitin, glycoproteins, and proteoglycans. As the first defense and a dynamic structure, PM integrity is maintained by mucin, chitin metabolizing enzymes, and peritrophins [31]. At 1 h, we observed *mucin* downregulation and *endochitinase* upregulation. This pattern may be consistent with infection strategies involving PM degrading factors, such as enhancin and ODV-E66, reported in other baculoviral systems that increase PM permeability and facilitate infection [22,23]. Subsequently, *peritrophin-1* upregulation at 24 h suggests a host-initiated repair response to PM damage, consistent with the PM-associated compensatory transcriptional changes reported in the AcMNPV-infected *Trichoplusia ni* midgut [18]. Following entry, viral intracellular transport relies heavily on the host cytoskeleton. AcMNPV utilizes proteins like *P78/83* to drive actin polymerization, while *ARIF-1* induces cortical actin rearrangement to assist barrier crossing [32,33]. The Rho GTPase signaling axis acts as a central hub for regulating cytoskeletal dynamics [34]. A related study in the *S. frugiperda* midgut also reported early ARP2/3 upregulation during primary baculovirus infection [27]. In our study, cytoskeletal genes were continuously upregulated from 12 to 24 h, whereas Graf was activated as early as 1 h and remained highly expressed, suggesting that cytoskeletal transcriptional reprogramming supports viral replication and budding. Finally, virions must breach the BL, a structural layer comprising *laminin*, *collagen IV*, and *nidogen*, along with co-factors such as *peroxidasin* and *hemicentin* [35,36,37,38,39]. Research indicates that baculovirus-encoded *vFGF* triggers a protease cascade involving matrix metalloproteinases and effector caspases, which remodels the BL to facilitate systemic viral dissemination [40]. Our results demonstrated a coordinated downregulation of multiple core BL components. This systemic transcriptional suppression, coupled with *matrix metalloproteinase* activation, suggests that SfMNPV may compromise BL structural integrity to create favorable conditions for systemic BV spread. In summary, the temporal and dynamic rearrangement of midgut barrier genes represents a key host factor for successful viral infection.

### 4.2. Coordinated Responses of Endoplasmic Reticulum Processing, Autophagy, and the Ubiquitin System

Baculoviruses establish infection in the host midgut epithelium, typically depending on the mediation of the host endoplasmic reticulum. Studies showed that the Sec61 complex in *Bombyx mori* acted as a pro-viral factor for BmNPV proliferation, and endoplasmic reticulum molecular chaperones including *calnexin*, *calreticulin*, and *endoplasmin* enhanced viral replication by maintaining the folding homeostasis of the viral envelope glycoprotein *GP64* [41,42]. Furthermore, the IRE1–X-box-binding protein 1 axis promoted viral proliferation, while *TER94*, a key driver of endoplasmic reticulum-associated degradation, was proven to directly participate in viral DNA replication and BV formation [43,44]. In the results of this study, these key genes in the endoplasmic reticulum were significantly upregulated at 12–24 h, suggesting that SfMNPV provides a stable intracellular environment for viral protein synthesis and budding through key factors in the endoplasmic reticulum. While the endoplasmic reticulum undergoes protein remodeling, host cells utilize autophagy and the ubiquitin system for resource recovery and protein turnover. In *B. mori*, BmNPV was proven to induce autophagy to significantly enhance viral replication and lethality [45,46,47]. The coordinated upregulation of autophagy-initiation factors *hamartin* and *tuberin-like* at 12–24 h further indicates that the virus attempts to activate the autophagy pathway to enhance infection. The ubiquitin-proteasome system is also essential for efficient baculovirus infection, as the proteasome inhibitor *MG-132* significantly reduced BmNPV budded virus yield and polyhedrin expression [48]. AcMNPV induces the accumulation of ubiquitinated proteins and the formation of aggregates in infected cells, accompanied by the linkage of proteasome and lysosome systems [49]. Further activation of *cathepsin B* marks the accelerated progression of degradation toward the lysosomal end, completing material degradation and cellular environment modification. In summary, this temporal expression pattern reflects the regulation of host endoplasmic reticulum processing, autophagy, and the ubiquitin dynamic system by SfMNPV, collectively constructing a metabolic microenvironment that efficiently supports viral proliferation and budding.

### 4.3. Dynamic Response Mechanisms of Detoxification Metabolism and Redox Homeostasis

The synergy between detoxification and antioxidant systems in the midgut is crucial for host resistance against viral infection. Our results showed that ABC transporters and UGT family genes were significant upregulation at 1 h. Research has shown that Phase II enzymes and Phase III transporters form an integrated defense network from detoxification to excretion. The UGT family can enhance the hydrophilicity of harmful substances through glycosylation [50]. *ABCB1* functions as a broad-spectrum efflux pump and *ABCC4* mediates the transport of metabolic conjugates [51,52]. Their upregulation suggested a host strategy to mitigate the accumulation of toxic components via enhanced membrane transport. Furthermore, *ABCD3*, localized to the peroxisomal membrane, might regulated lipid homeostasis [53]. The activation of the mitochondrial transporter *ABCB8* revealed the host’s early perception of mitochondrial stress and oxidative damage risk [54]. Recent studies on *B. mori* BmWh2 indicate that the function of some ABC members may extend beyond substance transport to involve the regulation of antiviral programs such as autophagy [55]. As the response progressed into the viral replication and budding phase from 12 to 24 h, GST family genes were continuously and significantly upregulated, which might have helped alleviate intracellular toxicity and clear lipid peroxidation products and ROS [56]. By 24 h, *SOD [Cu-Zn]*, its key molecular chaperone *CCS*, and *thioredoxin* were collectively and significantly upregulated, forming an antioxidant enzyme system. By scavenging excess reactive oxygen species (ROS) and repairing damaged proteins, these enzymes might have further maintained cellular homeostasis and mitigated oxidative damage caused by viral challenge [57,58]. Overall, the transcriptional response of the *S. frugiperda* midgut to SfMNPV revealing a systemic response from efflux detoxification to the maintenance of endogenous redox homeostasis.

### 4.4. Mechanisms of Midgut Epithelial Injury Repair and Regeneration

To address the barrier disruption and physiological stress caused by SfMNPV in the *S. frugiperda* midgut, the host may activate tissue repair strategies by triggering cell fate transitions. In insect defense mechanisms, shedding damaged epithelial cells and activating intestinal stem cell (ISC) proliferation for compensatory backfilling are key methods to prevent viral spread [59]. Research on mitochondrial apoptosis pathways indicates that *endonuclease* mediates nuclear DNA fragmentation during stress, while *programmed cell death protein* significantly accelerates the apoptotic process [60,61]. This study observed the synchronous upregulation of these two genes at 1 h, suggesting a possible early host response aimed at limiting viral expansion through local cell loss. Correspondingly, *apoptosis-inducing factor* induces chromatin condensation and mediates caspase-independent death upon stimulation [62]. The continuous high expression of this gene at 1, 12, and 24 h is consistent with mitochondrial mediated death signaling in the midgut. At 12 h, regenerative and repair signals began to emerge in the midgut cells. In the insect gut, *expanded* and *salvador* proteins function within the Hippo pathway to restrict tissue proliferation; thus, a decrease in their expression removes the inhibition of downstream effectors [63,64]. The significant downregulation of these two genes in this study suggests activation of ISC mediated regenerative proliferation. By 24 h, tissue remodeling appeared to involve the synergistic action of multiple pathways. Previous studies have shown that Wnt signaling is induced following intestinal injury, with its *frizzled* receptors driving directed stem cell differentiation [65,66]. The co-upregulation of these genes in this study is consistent with activation of midgut epithelial renewal and barrier reconstruction to counteract the damage induced by viral challenge. Regarding cellular metabolism, *LAMTOR2* mediates amino acid sensing and activates the mTORC1 pathway to drive cell growth [67]. Its enhanced expression may provide a molecular basis for the supply of energy and materials during the regeneration process. In summary, the *S. frugiperda* midgut showed a cascade response pattern to viral stress, progressing from cell death to regenerative repair and potentially contributing to the dynamic reconstruction of cellular barriers.

## 5. Conclusions

In summary, this study systematically characterized the temporal transcriptomic landscape of early midgut responses to SfMNPV challenge in *S. frugiperda*. By tracking the molecular evolution across the sampled time points, we identified significant dynamic remodeling of physical barrier integrity, cellular proteostasis, metabolic detoxification networks, and epithelial regeneration signaling. These patterns vividly reflect the intricate tug-of-war between viral manipulation of the host environment and the subsequent mobilization of multidimensional defense and compensatory mechanisms by the host. Accordingly, this work not only deepens our understanding of baculovirus pathogenesis but also provides critical leads for developing potent viral synergists and identifying novel molecular targets for pest management.

## Data Availability

The transcriptomic datasets in this study are available in the NCBI SRA database under accession number PRJNA1434403. The genome sequence data of the virus isolate used in this study have been deposited in NCBI GenBank under accession number PZ228810. The virus isolate is preserved in the China Center for Type Culture Collection under accession number CCTCC No. V202062.

## Supplementary Materials

The following supporting information can be downloaded at: https://www.mdpi.com/article/10.3390/insects17040401/s1, Figure S1: Quantitative real-time PCR validation, Error bars represent mean ± SD (n = 3). * *p* < 0.05, ** *p* < 0.01, *** *p* < 0.001; Table S1: Primer sequences of quantitative real-time PCR; Table S2: Quality analysis of sequencing data; Table S3: Summary of clean reads mapped to the reference genome.
