# Supplementary figures and images for: Time-Course Transcriptomic Analysis of Early Host Responses to Oral SfMNPV Challenge in Spodoptera frugiperda Larval Midgut
Source: Insects. 2026 Apr 8;17(4):401. doi: 10.3390/insects17040401 (PMC13116445; doi:10.3390/insects17040401)

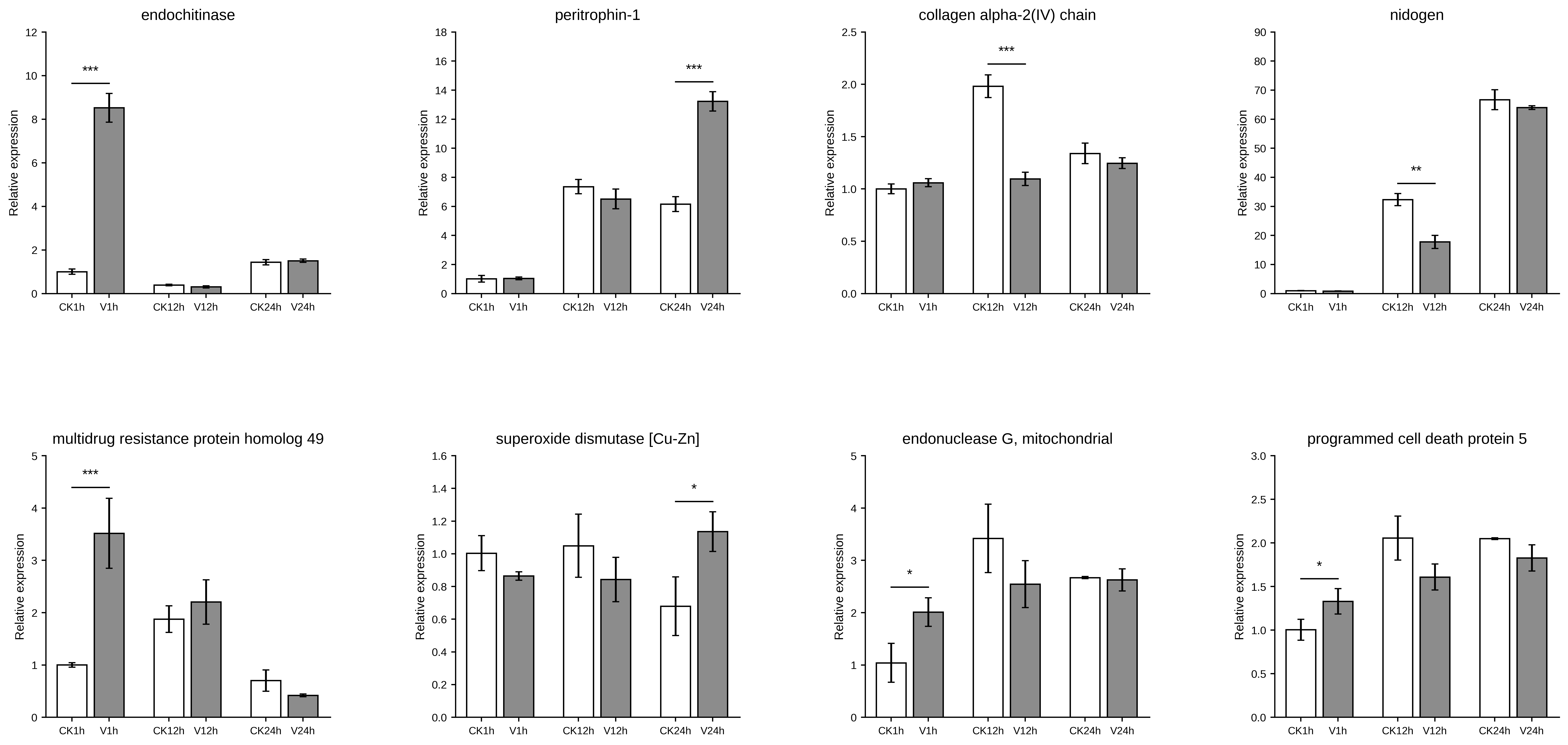

Supplement: Supplementary file 1 [file insects-17-00401-s001.zip › Figure S1. Quantitative real-time PCR validation..png]
